# Measuring the Height of a Tree

**Published:** 1885-02

**Authors:** 


### MEASURING THE HEIGHT OF A TREE.

There is a very simple way of measuring the height of a tree which can be practiced by any one on a sunny day or in bright moonlight. All the apparatus that is necessary is a straight stick, of any length. Draw a circle with a radius (half the diameter) of a little less than the length of the stick. This will be done by holding one end of the stick, say two inches from its end, and moving the other end around, making the circle with a knife or a chip. Then place the stick in the ground exactly in the center of the circle, perfectly upright, and press it down until the height of the stick is exactly the same as the radius of the circle.

When the end of the shadow of the stick exactly touches the circle, then also the shadow of the tree will be exactly in length the same measurement as its height. Of course, in such a case, the sun will be at an exact angle of forty-five degrees.

Measurements of this character can be best effected in the summer, when the sun is powerful, and has reached to a good height in the heavens, and when the trees are clothed with living green, so as to cast a dense shadow.

To many to whom this idea may not have occurred, it might be made annually a matter of interest thus on warm summer days to take the height of prominent trees, and so to compare growth from year to year.—*Youth's Companion.*

---

The death-rate of Russia is the highest in Europe. This is attributed to the paucity of medical men and the habits of the rural population. According to late returns the average duration of life is only 26 years, and the mortality among infants is frightful. More than 60 per cent. of infants die before they reach their fifth year, and nearly 2,000,000 children perish every year. Of 8,000,000 boys, only 3,770,000 attain the age of military service—that is to say, their twenty-fifth year, and of these at least 1,000,000 are found, by reason of shortness of stature and weakness of body, to be unfit for military duties.

---

Hall's Journal of Health will be supplied in quanties to suit the trade and newsdealers by the American News Company, who are general agents for the publication.

---

Subscribers will confer a favor by making their remittances by check when practicable, or in one and two-cent stamps.

